# Engineering Moxifloxacin-Encapsulated Liposome-Enriched Alginate Hydrogel Films

**DOI:** 10.3390/gels11060448

**Published:** 2025-06-11

**Authors:** Ismail Bal, Meltem Macit, Ali Alasiri, Onur Cem Namli, Muhammad Sohail Arshad, Zeeshan Ahmad, Gulengul Duman, Israfil Kucuk

**Affiliations:** 1 Institute of Nanotechnology, Gebze Technical University, Kocaeli 41400, Turkey; i.bal2019@gtu.edu.tr; 2 Vocational School of Health Services, Istanbul Okan University, Istanbul 34959, Turkey; 3 Department of Pharmaceutical Technology, Faculty of Pharmacy, Yeditepe University, Istanbul 34755, Turkey; meltem.macit@yeditepe.edu.tr (M.M.); gduman@yeditepe.edu.tr (G.D.); 4 Department of Pharmaceutics, College of Pharmacy, Najran University, Najran 55461, Saudi Arabia; aalasiri@nu.edu.sa; 5 Department of Mechanical Engineering, Faculty of Engineering, Yeditepe University, Istanbul 34755, Turkey; onur.namli@yeditepe.edu.tr; 6 Department of Pharmacy, Bahauddin Zakariya University, Multan 60800, Pakistan; 7 Leicester School of Pharmacy, De Montfort University, Leicester LE1 9BH, UK; zahmad@dmu.ac.uk

**Keywords:** moxifloxacin, liposome, T-shaped microfluidic junction device, hydrogel, prolonged drug delivery

## Abstract

In the present study, we developed a moxifloxacin (MXF)-encapsulated liposome-enriched alginate nanocomposite hydrogel coating. MXF was encapsulated in soy lecithin (SL:MXF:2:1) via the probe sonication method with an average efficiency of 80%. Two different manufacturing methods, including a micropipetting and a T-shaped microfluidic junction (TMJ) device technique, were used to incorporate the MXF-encapsulated liposomes into hydrogel matrices and layered as a coating on polymeric substrate material. Drug encapsulation and its incorporation into the hydrogel matrix significantly enhanced its stability and facilitated a prolonged drug release profile. A relatively rapid drug release was observed in the MXF-encapsulated liposome-loaded polymeric particulate layer developed via the micropipetting than the TMJ device technique. The findings confirmed sustained drug release behavior due to a hydrogel particulate structural uniformity conferred by the micromachine device, TMJ. Thus, these nanocomposite hydrogel coatings achieved can serve as a promising candidate for the treatment of ophthalmic or mucosal membrane infections.

## 1. Introduction

Over the past few decades, nanoparticle-based systems (micelles, dendrimers, liposomes, or niosomes) have been evaluated as an alternative for treating ophthalmic diseases and mucosal membrane infections, as they may offer reduced irritation and improved bioavailability [1]. Many types of nanoparticles, including liposomes and polymeric nanoparticles, have been developed to enable the precise and efficient delivery of drugs to targeted sites [2]. Liposomes, also known as lipid vesicles, are microscopic vesicles, with sizes ranging from 10 nm to 1 µm, featuring an aqueous core enclosed by one or more concentric lipid bilayers [3,4]. They are advantageous due to their unique properties, including non-toxicity, biocompatibility, and ability to accommodate both hydrophilic and hydrophobic drugs, and their membrane-like natural structure [5,6].

Moxifloxacin (MXF), a fluoroquinolone antibiotic, is commonly utilized in the management of ocular infections, including postoperative endophthalmitis [7,8,9]. Studies conducted to determine the efficacy of MXF in the treatment of post-surgical infections have demonstrated that MXF causes minimal toxicity in the anterior segment or cornea [10,11]. Furthermore, MXF provides 2–3 times higher concentration in the aqueous humor as compared to other fluoroquinolones [12,13]. Several studies in the literature have investigated the use of MXF-loaded liposomes for ocular applications [14,15].

Liposomes are prepared using different techniques, including thin-film hydration, solvent depletion, solvent injection, reverse-phase evaporation [16], and probe sonication [17,18,19]. Among these, probe sonication is preferred as it provides a uniform size distribution. Furthermore, better encapsulation is expected from these liposomes. Although the incorporation of a drug into liposomes protects enzymatic hydrolysis in the body, they can also be incorporated into polymer-based hydrogels to increase their stability and enhance their controlled drug delivery [20,21,22]. Composite hydrogel systems have been reported to offer extended drug release while preserving the stability of liposomes and the bioactivity of the drug [23,24]. Natural polymers for hydrogel production, such as cellulose, hyaluronic acid, chitosan, collagen, and alginate, are preferred because they are economical, non-toxic, biocompatible, and biodegradable [25,26,27]. Sodium alginate, a Food and Drug Administration (FDA)-approved polysaccharide, has the ability to form hydrogel via cross-linking through ionic gelation in the presence of calcium chloride [28,29,30,31]. The key feature of alginates is their ability to form water-resistant structures that benefit sustained release of encapsulated drug substances [32,33].

Incorporation of liposomes into the hydrogel matrix has been performed using various techniques, including emulsion, suspension polymerization, bulk polymerization, solution polymerization/cross-linking, micropipetting, electrospraying, 3D printing, and microfluidics. Microfluidics is a beneficial technique as it is a water-based processing routine, suitable for hydrophilic hydrogel-forming polymers and ensuring liposomal nanoparticles’ monodisperse distribution [34,35,36,37,38].

We aimed at achieving monodispersed MFX-enriched liposomes in the hydrogel matrix of predetermined size distribution, which remits a predictable drug release profile. Herein, we developed MXF-encapsulated liposome-enriched alginate nanocomposite hydrogel coatings by utilizing the following two different techniques: micropipetting and the TMJ device. Particle size and distribution, polydispersity index, zeta potential, and encapsulation efficiency values were assessed for MXF-encapsulated liposomes. Fluorescent microscopy images were captured to monitor MXF-encapsulated and non-encapsulated liposomes. Fourier-Transform Infrared Spectroscopy (FTIR) and Scanning Electron Microscopy (SEM) analysis of the resultant MXF-encapsulated liposomes in order to unveil their chemical compositions and morphologies were determined, respectively. FTIR, SEM, and Energy-Dispersive Spectroscopy (EDS) analysis and drug release behavior were investigated for MXF-encapsulated liposome-enriched alginate nanocomposite hydrogels. The encapsulation of MXF and its incorporation into a hydrogel coating layer resulted in an improvement in the controlled and prolonged drug delivery features of these alginate-based nanocomposite coatings. These findings demonstrate the potential of MXF-free and MXF-encapsulated liposome-enriched alginate nanocomposites as a promising candidate for treating membranous drug delivery.

## 2. Results and Discussion

MXF-encapsulated liposome-enriched alginate nanocomposite hydrogel coating constructs were successfully deposited on a polymeric substrate material.

### 2.1. MXF-Free and MXF-Encapsulated Liposome Formation by Probe Sonication

**Figure 1:**
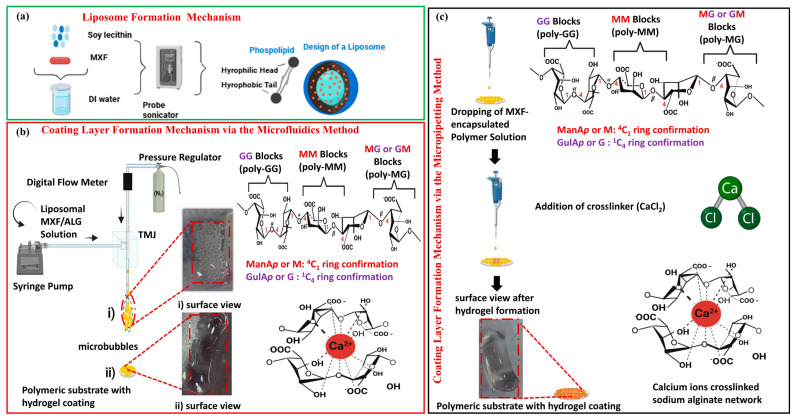
Schematic representation of (**a**) the liposome formation mechanism and MXF-encapsulated liposome-enriched alginate hydrogel coating formation via using (**b**) the TMJ device technique and (**c**) the conventional micropipetting methods. (**i**) coating microbubbles on the polymeric substrate material with a layer form before gelation, and (**ii**) a hydrogel appearance on the polymeric substrate material after cross-linking alginate with CaCl_2_ can be seen in the inserted captures (generated using biorender.com).

The MXF-loaded liposomes were prepared using a probe sonicator, as illustrated in Figure 1a. When phospholipids comprise one or two fatty acid groups, which are dissolved in water, they spontaneously assemble into flat bilayers. With the effect of high-frequency sound waves applied by probe sonication, the hydrophobic tails orient inward, facing one another, while the hydrophilic heads are exposed to the aqueous environment. Liposomes are generated through the disintegration of these double lipid layers, subsequently reassembling into spherical structures. Typically, liposomes can maintain their physical stability for a defined duration, a phenomenon referred to as “kinetic stability.” However, over time, the particles tend to agglomerate, resulting in an increase in particle size [17]. The liposome solutions obtained at different amplitudes and times were freeze-dried and prepared for characterization. Liposomes with average particle sizes ranging from 150 ± 1.28 nm to 186 ± 1.06 nm were successfully obtained.

### 2.2. Characterization of Freeze-Dried Liposomes

**Figure 2:**
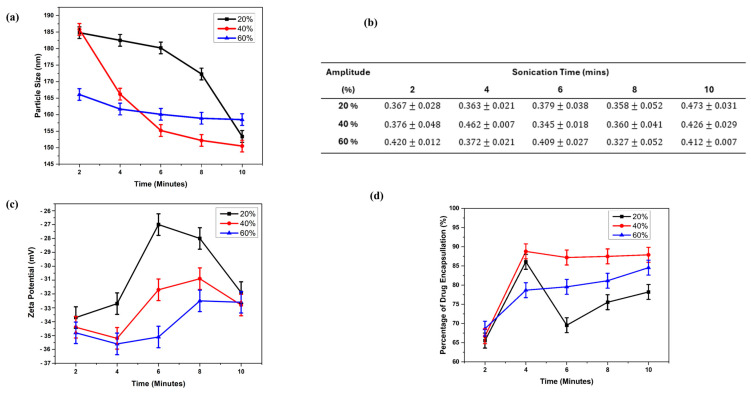
(**a**) Particle size, (**b**) PDI, (**c**) zeta potential, and (**d**) EE values for the SL:MXF:2:1 liposome (soy lecithin + mxf) produced by the probe sonication at room temperature conditions with different amplitudes (20%, 40%, and 60%) and sonication times (2 min, 4 min, 6 min, 8 min, and 10 min).

Figure 2 shows results of MXF-free or encapsulated liposome nanoparticles (e.g., 15 various formulations) produced by the probe sonication technique. Figure 2a shows the particle size of the liposome for 15 different liposome nanoparticle formulations, with particle sizes ranging from 150 ± 1.28 nm to 186 ± 1.06 nm. This particle size range is acceptable for ocular drug delivery [18]. As amplitude values of sonication were kept constant and its duration was increased from 2 min to 10 min, the diameter of the resultant liposomes decreased. Liposomal size distribution was minimally affected by the nature of the drug substances. Nevertheless, this observation requires further validation, as the authors have studied the particle geometries in the presence or absence of MXF.

Figure 2b shows the polydispersity index (PDI) values for various liposome formulations. For liposomes, the PDI value is a crucial parameter that influences the bulk characteristics, stability, performance, and appearance of the product [39]. This table indicates the values of PDI of various liposome formulations, which are between 0 and 1. These results confirm the uniformity of the particles [40]. The values are close to zero, indicating narrow size distribution. Thus, each liposome produced may have the potential to encapsulate a similar amount of drug. Figure 2c shows the zeta potential values of liposomes. The resultant zeta potential values of the liposome are described in Figure 2c, which shows a surface charge value change from −35.5 ± 0.99 mV to −30 ± 0.66 mV (except the value of the sample L3 was −27 ± 1.17 mV). It is pertinent to mention here that liposomes with zeta potentials of greater than +30 mV or less than −30 mV are considered highly stable due to the sufficient repulsive force, which brings about physical colloidal stability. The results confirm that all liposome formulations exhibit good stability [41]. Figure 2d shows encapsulation efficiency (EE) for different liposome formulations. EE values observed between 65.55% and 88.8% indicate good encapsulation efficiency [19]. This can also be regarded as the drug loading capacity, which is directly related to the EE. The hydrogel coating contains a quantity of drug corresponding to the EE percentage. The EE can be increased by using organic solvents like ethanol and chloroform [15]. However, using a solvent other than water was not preferred due to the sensitive nature of the eye. Experimental results have shown that the probe sonicator can be used to produce organic solvent-free liposomes.

**Figure 3:**
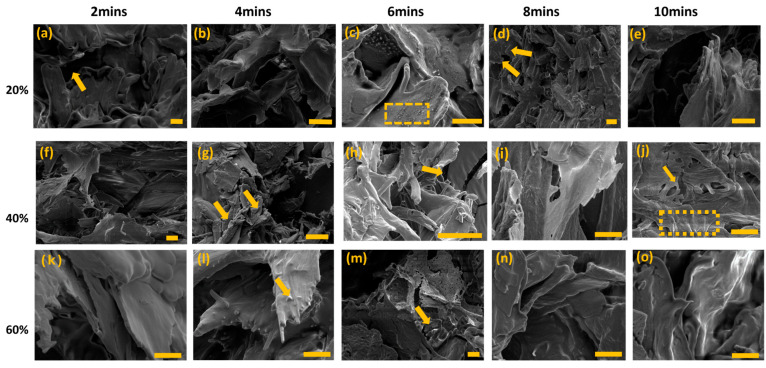
(**a**–**o**) SEM images of the SL:MXF:2:1 liposomes (soy lecithin + MXF) produced by the probe sonication technique by adjusting its amplitudes from 20 to 60% and duration of sonication process between 2 and 10 min (The scale bars represent 5 µm. The images were captured at different magnifications as follows: (**b**) at 1000×; (**a**,**d**,**f**–**h**,**m**) at 2000×; and (**c**,**e**,**i**–**l**,**n**,**o**) at 5000×.

Figure 3 shows SEM micrographs captured for various liposome formulations. In Figure 3, SEM images show the tendency of small dot-like structures where individual particles can be distinguished in the light grey areas. Arrows and dash-lined squares indicate the areas where these structures are most prevalent. It is evident that rather than being encased in a spherical structure, the MXF drug is encapsulated in tiny granular particles. Given that this research emphasizes the natural production of the matrix intended for ocular incorporation, only deionized (DI) water was employed as the solvent. This choice can be attributed to the observation that the resulting liposomes do not exhibit a spherical morphology, which can be seen in Figure 3.

Further confirmation of the drug–lipid interaction FTIR spectra of the liposomes achieved was measured and compared, as seen Figure S1. The characteristic peaks in pure MXF spectra (Figure S1) were represented by the stretching vibrations C=O at 1735 cm^−1^, N-H at 2949 cm^−1^, and O-H at 3327 cm^−1^. Soy lecithin showed peaks at 3671 cm^−1^ and 1703 cm^−1^ for the O-H and C=O stretching frequencies, respectively. Stretching vibrational peaks at 1170 cm^−1^ and 1062 cm^−1^ can be attributed to the C–O and C–C bonds. The primary absorption bands of lecithin and MXF hydrochloride were identified in the FTIR spectra of the liposomes produced, exhibiting no significant alterations (Figure S1; L1–L15). This observation suggests that there is no significant potential chemical interaction between the drug and the lipid components.

**Figure 4:**
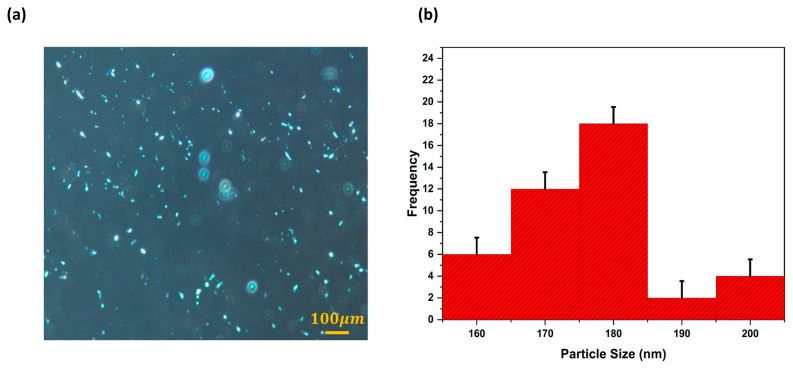
(**a**) Fluorescent/polarized microscope caption and (**b**) size distribution of the L7-labeled liposome nanoparticle produced by the probe sonication method. Data are presented as mean ± SD (approximately 300 particles per ten images were evaluated).

To track the encapsulated drug and provide a better understanding of liposomes, fluorescent/polarized microscopy was performed. The captured MXF-encapsulated liposomes and particle size distribution are demonstrated in Figure 4. The shiny and spherical multivesicular structures can be clearly seen in Figure 4. Free (non-encapsulated) particles are also visible. Particle size distribution analysis was also performed by evaluating approximately 300 particles per 10 images, and the average particle size was calculated to be 174 ± 0.566 nm using ImageJ software Version 1.46j. The results are consistent with the data obtained from dynamic light scattering (DLS). Due to the luminescent properties of MXF, imaging and localization of the drug using fluorescence microscopy were facilitated. Fluorescent microscopy made the layers of the liposome structure apparent. Multivesicular vesicles contain non-concentrically ordered smaller internal vesicles [42].

**Figure 5:**
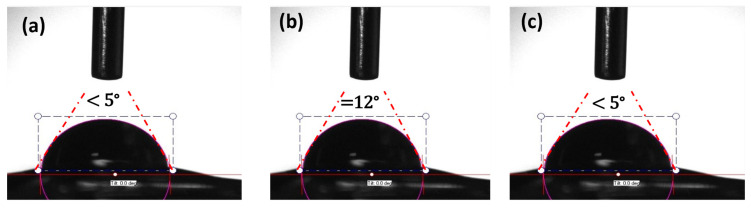
CA Measurements of (**a**) pristine sodium alginate, (**b**) L7-labeled liposomes, and (**c**) MXF-encapsulated liposome-enriched alginate nanocomposite hydrogels.

Figure 5 shows contact angle (CA) values of pure sodium alginate, MXF-encapsulated liposome (L7), and MXF-encapsulated liposome-enriched alginate nanocomposite hydrogel. CA measurements resulted in ‘<5°, =12°, and <5°’, respectively. CA defines a measurement that quantifies the wetting of a solid by a liquid. A low CA indicates high wettability, while a high CA suggests hydrophobic characteristics. CA measurement offers valuable data for optimizing surface treatments and understanding interactions at the micro- and nano-scales. The measurements of CA were smaller than 90 degrees, which indicates hydrophilicity. The incorporation of hydrophilic drugs into hydrogel matrices often results in a burst release, leading to a short-term drug release profile. Thus, encapsulating hydrophilic drugs within liposomes facilitates controlled and adjustable drug release [43].

Although other liposomes were also suitable for incorporation into hydrogel, the liposome nanoparticle labelled L7 was selected as the most suitable formulation, as it has the desired particle size, showing the best monodisperse distribution in the sample.

### 2.3. Characterization of Hydrogel Nanocomposites

#### 2.3.1. Microbubble Formation

MXF-encapsulated liposome-enriched alginate nanocomposite hydrogel coating constructs have been obtained via the TMJ device when a continuous, steady stream of bubbles was initially achieved, as seen in Figure 1. The alginate-based polymer solution flows at a constant rate, while inert gas nitrogen (N_2_) is introduced at a constant pressure of 0.5 bar, resulting in necking within the outlet channel. The interaction of the non-mixing dispersion phase (liquid) and the continuous phase (gas) generates distinct shear stresses, resulting in the formation of micron-sized bubbles. The liquid or gas phase leaves the fluid and adheres to the other fluid phase in a microliter volume; this frequently takes the elucidation of bubbles with spherical shapes to stabilize interfacial instability.

The generated bubbles flow down through the exit channel and are collected on a polymeric substrate material. By passing liquids through the micron-sized channels, microbubbles can be produced in uniform distribution and desired sizes. Under an optical microscope, the resultant droplets were approximately 180 μm in diameter. A microbubble cluster can be seen on the substrate surface. To finalize the formation of the alginate-based nanocomposite hydrogel coatings, the cross-linking agent calcium chloride was added to the bubbles and subsequently left for 1 h to perform ionic cross-linking of alginate polymeric microbubbles at +4 °C [37].

#### 2.3.2. Fourier-Transform Infrared Spectroscopy (FTIR) Method

**Figure 6:**
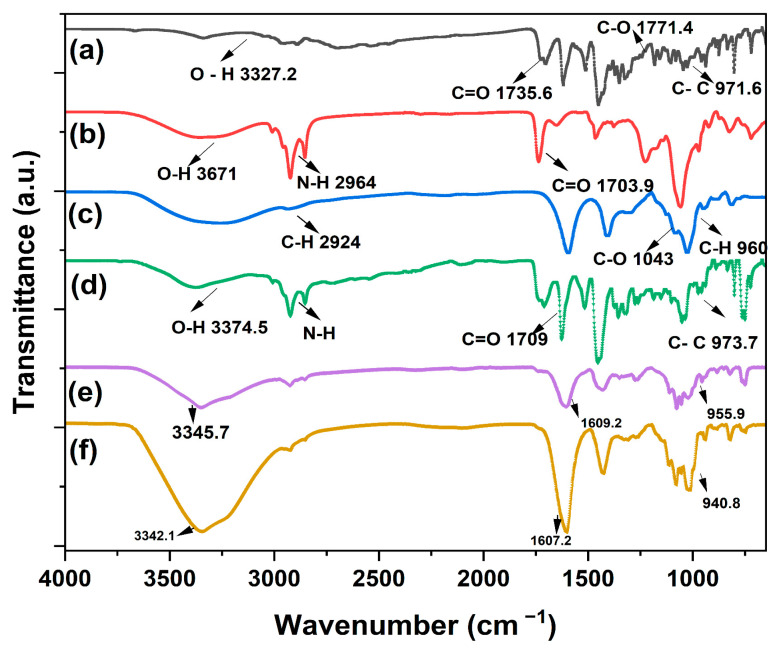
FTIR spectra for (**a**) pristine MXF, (**b**) pristine lecithin, (**c**) pristine alginate, (**d**) SL:MXF:2:1 liposomes (soy lecithin + MXF) L7 liposomes, and the resultant MXF-encapsulated liposome-enriched alginate nanocomposite hydrogel coating constructs via (**e**) the TMJ device and (**f**) the micropipetting method.

The chemical composition of the pristine MXF, pristine lecithin, and alginate; MXF-encapsulated liposome and MXF-encapsulated liposome-enriched alginate nanocomposite hydrogels produced via the micropipetting technique; and the TMJ device method was determined using FTIR spectroscopy. The results can be seen in Figure 6.

During spectral analysis, the noticeable absorption peaks that MXF displayed were readily apparent, including a peak at 3327 cm^−1^ for the OH stretch. Additionally, symmetrical and asymmetrical C-H stretch are represented by the absorption peak at 2924 cm^−1^, C=O stretch by 1609 cm^−1^, C=C stretch by 1620 cm^−1^, 1517 cm^−1^, and 1453 cm^−1^, and C-H bending by the peak at 940 cm^−1^. The FTIR spectrum of the MXF-loaded liposome-incorporated hydrogels (for both micropipette- and microfluidics-produced) demonstrated the respective absorption peaks presented by MXF, corresponding to C=O stretching. Respective absorption from the extracted lecithin had an N (CH_3_)_3_ group bond at 960 cm^−1^, and P =O at 1231–1232 cm^−1^ [44,45]. Infrared absorption measurements were conducted to acquire insights into the chemical properties of both pure ingredients and the alginate-based nanocomposite hydrogel coating constructs, as well as the bonding and interactions between these formulations.

#### 2.3.3. Scanning Electron Microscope (SEM) of Hydrogel Nanocomposites

**Figure 7:**
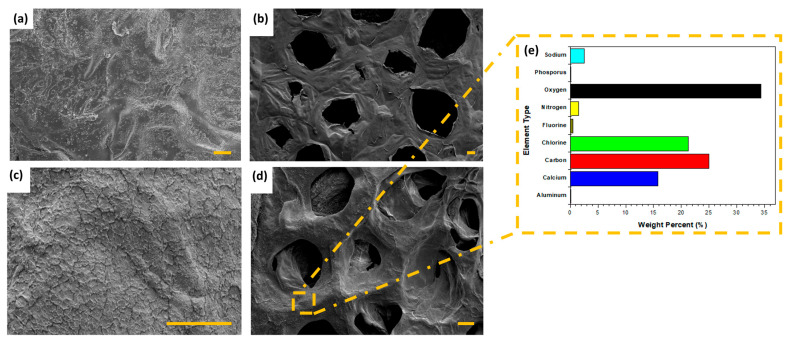
SEM Images of the produced alginate-based hydrogel coating constructs without MXF by using (**a**) the micropipetting method and (**b**) the TMJ device technique; MXF-encapsulated liposome-enriched alginate nanocomposite hydrogels produced by (**c**) the micropipetting method and (**d**) the TMJ device technique; and (**e**) EDS analysis of the MXF-encapsulated liposome-enriched alginate hydrogel coating constructs (The scale bars represent 40 µm. The images were captured at different magnifications as follows: (**b**) at 125×, (**a**,**d**) at 250×, and (**c**) at 1000×.

Figure 7 shows the difference between the morphological structure of using different production techniques for producing hydrogels as well as the distribution of MXF-encapsulated liposomes through the hydrogel. Surface morphology may serve as an indicator for explaining drug release behavior; a porous structure results in prolonged release due to the increased surface area. Thus, the surface morphologies of MXF-free and MXF-encapsulated liposome-enriched alginate nanocomposite hydrogels produced with two different routes, including the micropipetting technique and the TMJ device method, are presented in Figure 7. The influence of the hydrogel production method on its morphology is evident. Specifically, a layered structure was observed in hydrogels produced using the micropipetting technique, whereas the TMJ device route effectively resulted in the formation of the monodisperse porous structure. Figure 7a,b show the micropipetting technique and TMJ device-driven MXF-free hydrogel coating constructs having dense outer surfaces. To complete drug tracking and compare them by using SEM micrographs, drug-included hydrogel images were captured (see Figure 7c,d). The small particles that appear to be scattered on the surface are presumed to be MXF-encapsulated liposomes, as shown in Figure 7c,d. In comparison, a specific quantity of crystalline substances is embedded within the surface texture due to the drug MXF. The same behavior was observed on model ibuprofen distributed in the hydrogel thin film [46].

In Figure 7e EDS, the result of the MXF-encapsulated liposome-enriched alginate nanocomposite hydrogels can be seen. The relative ratio of that element can only be ascertained for each location on the sample surface by choosing the X-rays that are represented by the peaks of the element of interest and counting just the X-rays in the EDS detector. The presence of liposomal MXF liposomes both inside and outside the pores can be seen in the polymer network structure. In Figure 7e, the fluorescence intensity is reported as 0.40% and the phosphate content as 0.02%, indicating the successful incorporation of both liposomes and MXF into the hydrogel matrix. This result indicates that the presence of fluorescence and phosphate amounts in the sample confirms the incorporation of both liposomes and MXF, as well as their successful dispersion within the hydrogel matrix.

#### 2.3.4. Investigation of Swelling Profile

**Figure 8:**
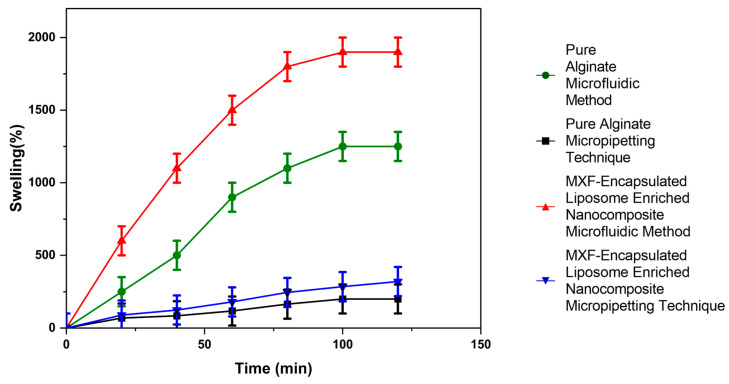
Swelling ratio values of MXF-free and MXF-encapsulated liposome-enriched alginate nanocomposite hydrogels.

Figure 8 indicated that the percentage swelling ratios of both MXF-free and MXF-encapsulated liposome-enriched alginate nanocomposites produced by two different methods, micropipetting and TMJ, at different times up to 120 min. The swelling index, also known as the swelling ratio, is a key feature of hydrogels that defines their capacity for water absorption. This characteristic, in turn, influences the release behavior of encapsulated drug molecules. The swelling process for nanocomposite hydrogels includes the following three main steps: water molecules adsorb to the surface of the nanocomposite hydrogel; the weakening of intermolecular and hydrogen bonding interactions occurs as water molecules migrate into the cross-linking network, leading to the subsequent expansion of the polymeric chains; and, finally, the formation of porous structures facilitates the penetration of additional water molecules. Figure 8 shows that MXF-encapsulated liposome-enriched alginate nanocomposite hydrogel produced by the TMJ method demonstrated a nearly comparable swelling ratio at room temperature in DI water media. Nanocomposite hydrogels were characterized by a maximum swelling index of approximately 2000% ± 30 in the initial 80 min and subsequently gradually increasing until 120 min.

#### 2.3.5. Mechanical Properties

**Figure 9:**
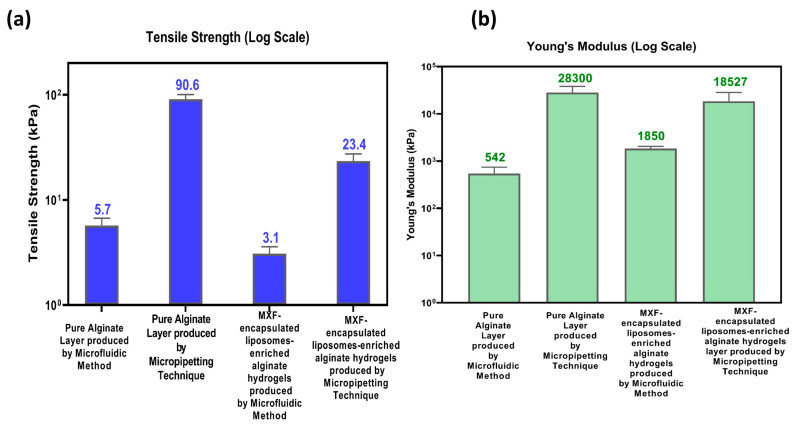
(**a**) Tensile strength and (**b**) Young’s modulus values for MXF-free and MXF-encapsulated liposome-enriched alginate nanocomposite hydrogels. Data are presented as mean ± SD with an estimated 5% error margin.

Figure 9 shows the mechanical properties of MXF-free (pure alginate) and MXF-encapsulated liposome-enriched alginate nanocomposite hydrogels produced by the following two different methods: TMJ and micropipetting. The tensile strength values for hydrogels are displayed in Figure 9a. The lower tensile strength observed in hydrogels synthesized by TMJ can be attributed to the presence of a microbubble structure. Microbubbles create weak interactions within the hydrogel matrix that can act as stress concentrators, making it easier for the material to break under tension, reducing the effective load-bearing area, and disrupting the continuity of polymer chains, contributing to the lower tensile strength. However, the tensile strength increases with the addition of MXF-encapsulated liposomes in both pure alginate and alginate nanocomposite hydrogels. The increase in tensile strength is because the liposomes enhance the cross-linking within the hydrogel, providing additional structural support and improving the material’s ability. Figure 9b shows the Young’s modulus values for the selected hydrogel samples. These values are consistent with the tensile strength results. Hydrogels fabricated using the micropipetting technique exhibit higher Young’s modulus values for MXF-free and MXF-encapsulated liposome-enriched alginate nanocomposite hydrogels. The hydrogels produced using the TMJ device are more elastic because they contain microbubbles that can compress and expand, making the material more flexible.

#### 2.3.6. In Vitro Moxifloxacin Release of the Alginate-Based Nanocomposite Hydrogel Coating Composites

**Figure 10:**
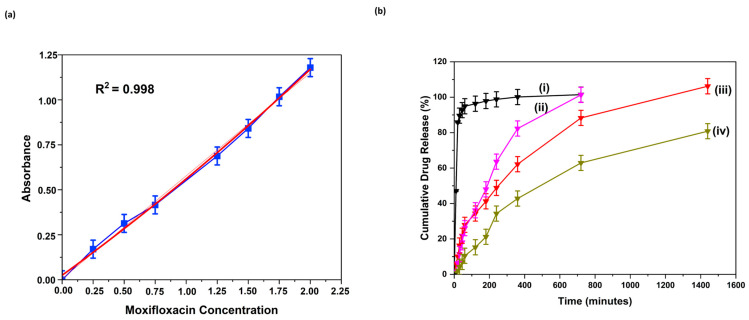
(**a**) Calibration curve of the drug MXF at different concentrations at room temperature environment (Absorbance is represented by the blue line, while the red line corresponds to its polynomial fitting). (**b**) In vitro cumulative drug release behavior of MXF-encapsulated liposome nanoparticles layered coating form and MXF-encapsulated liposome-enriched alginate nanocomposite hydrogel coating constructs produced by the micropipetting method and the TMJ device technique. (**i**) MXF-encapsulated liposome (micropipetting technique), (**ii**) MXF-encapsulated liposome (microfluidic method), (**iii**) MXF-encapsulated liposome-enriched alginate hydrogels (micropipetting technique), and (**iv**) MXF-encapsulated liposome-enriched alginate hydrogels (microfluidic method).

The amount of drug released from MXF-encapsulated liposomes and nanocomposite hydrogel produced using a micropipetting technique and TMJ device method was determined by a spectroscopic method at 290 nm. The regression equation was evaluated as $y=0.5723\;x+0.0057\;(R^{2}=0.998)$, as shown in Figure 10a.

Herein, the cumulative drug release from different formulations was measured under physiological conditions (in DI water at 37 °C). Figure 10b compares the time-dependent cumulative release of MXF from drug-loaded liposome and MXF-encapsulated liposome-enriched alginate nanocomposite hydrogel coating layers produced by two different methods. The dissolution profiles depicted herein in Figure 10b show the released amount of MXF in total. The hydrogels produced by using the TMJ device technique demonstrated a slower drug release profile compared to those fabricated with the micropipette method. The liposome formulation produced with the micropipetting (black line in Figure 10b) showed ‘burst release’, meaning it released almost all the drug within a few hours. The same liposome formulation produced via the TMJ device method (pink line in Figure 10b) exhibited relatively the prolonged drug release compared to the equivalence, thus pointing out the effects of the production method on release behavior. When comparing the release behavior of MXF-encapsulated liposome-enriched alginate nanocomposite hydrogels (red and dark green lines in Figure 10b) based on the production method, it is evident that the porous structure achieved through the TMJ device technique governs the prolonged drug release profile. The incorporation of MXF-encapsulated liposomes into the hydrogel network significantly manipulated drug release; however, it was observed that the duration required to release the entire drug content was extended when compared to formulations consisting solely of MXF-encapsulated liposomes. This can be attributed to maintaining the stability of the liposomes dispersed within the hydrogel. The diffusion of the drug through the polymeric matrix is hindered by strong interactions that arise from the mixing of the two macromolecules. The incorporation of MXF-encapsulated liposomes into the hydrogel network introduces cross-linked chains that serve as a barrier, thereby restricting drug diffusion. Upon the penetration of water molecules into the nanocomposite hydrogel, polymer degradation occurs, facilitating the diffusion of drug-loaded liposomes that are homogeneously distributed throughout the hydrogel’s polymeric network. Once in the polymeric matrix, water molecules interact with the encapsulated drug molecules and dissolve them [47]. The water-soluble drug molecules determine the drug release from the nanocomposite hydrogel.

Drug release profiles of all formulations were analyzed by applying different dissolution models, including first-order, Higuchi, Korsmeyer–Peppas, and Hixson–Crowell (*n* = 3). Two types of release patterns were observed in the system, which can be classified as follows: (i) the first 60 min (Table 1) and (ii) the period between 120 and 1440 min (Table 2). The first hour gives drug release following the Korsmeyer model, suggesting gel swelling and erosion diffusion. Afterward, there is a Hixson–Crowell model indication of spherical particle deshaping release. On the basis of the R-square (R^2^) value, the MXF liposome formulation prepared using the micropipetting method followed first-order kinetics. MXF liposomal formulation prepared by the TMJ approach followed the Korsmeyer–Peppas model with an n value of 0.467, which suggested that the release was governed by a diffusion mechanism. Nanocomposite hydrogel prepared using the micropipetting approach followed the Hixson–Crowell model. The release profile of the nanocomposite formulation prepared by the TMJ method followed first-order kinetics.

## 3. Conclusions

MXF-free and encapsulated liposome nanoparticle-enriched alginate hydrogel coatings on polymeric substrate material were formed successfully by using the following two different techniques: the micropippetting and TMJ device techniques. MXF encapsulation in liposome nanoparticles is achieved successfully by using the probe sonication method to incorporate into alginate hydrogel coating constructs for deposition onto the polymeric substrate material. Optimum particle features 155 ± 0.041 nm particle diameter with a PDI value of 0.462%) of the MXF-encapsulated liposomes (labeled as L7) were determined and assessed to apply for the hydrogel coating material. Preparation of MXF-encapsulated liposome-enriched alginate nanocomposite hydrogel coating constructs by the TMJ device method results in a three-dimensional network porous structure with uniform pore size distribution. Structural features directly affect the drug release characteristics. The encapsulation of MXF into liposomes, as well as the dispersion of these liposomes within an alginate hydrogel matrix, directly influenced the drug release behavior. The concentration vs. time profile indicates linear characteristics, with an R^2^ value of 0.998. The mucoadhesive function of the gelling agent will permit increased residence time of nanocarriers at the site of injury.

## 4. Materials and Methods

### 4.1. Materials

The encapsulation excipient drug, MXF hydrochloride, was received as a gift from Sanovel Pharmaceutics Company, İstanbul, Turkey. L-α-Phosphatidylcholine (from soybean, Type IV-S) used for liposome production, polyethyleneglycol-40 stearate (PEG-40S, with a density of 1300 kg·m^−3^), analytical grade sodium alginate (with a viscosity of 16.94 mPa.s) employed as a hydrogel coating layer, and calcium chloride dihydrate for physical cross-linking were purchased from Sigma-Aldrich, Rockville, MD, USA. Inert gas nitrogen (N_2_) was procured from Linde Gas (99.9% purity, Kocaeli, Turkey). A Millipore device was used to filter distilled water (Arium 611UV, Sartorius, Lower Saxony, Germany).

### 4.2. Preparation of Moxifloxacin-Encapsulated Liposomal Alginate Hydrogel Nanocomposite Coatings

MXF and soy lecithin were sonicated by the probe sonication method for encapsulation of the drug into the liposome nanoparticles. These resulting dispersions were freeze-dried for their characterization. Next, by using the MXF-encapsulated liposomes mixed with alginate polymer to prepare a coating solution. Subsequently, the coating was deposited onto polymeric substrate material by using two different approaches: a micropipetting method and a TMJ device technique. Characterization procedures of the resultant hydrogels were conducted to confirm whether the hydrogel coating layers were successfully formed to facilitate controlled drug release as anticipated.

#### 4.2.1. Preparation of Only MXF Drug Solution and MXF-Encapsulated Liposomal Nanoparticles

First, the MXF drug solution was prepared with a concentration of (1% *w*/*v*), which was dissolved in 10 mL of DI water by using a magnetic stirrer. Then, MXF-encapsulated liposome nanoparticle preparation was delivered by using the probe sonication method (Nanografi Nano Technology, Ankara, Turkey). In this liposome nanoparticle preparation technique, 10 mg of MXF-free (or blank for control), 10 mg of MXF, and 20 mg of soy lecithin polymer were dissolved in 10 mL of DI water with a ratio of SL:MXF:2:1, and subjected to ultrasonic force for encapsulation.

Processing parameters in probe sonication for liposomal nanoparticles were adjusted for ranges from 2 to 10 min with 2-min increments and various types of amplitude values (20%, 40%, and 60%) (Nanografi Nano Technology, Ankara, Turkey). Time and amplitude values were optimized to prevent excessive heating to protect drug stability. Moreover, the parameters also influence the EE and average particle size. Then, the resulting solutions were freeze-dried to make them ready for characterization (at −55 °C, 1 mbar for 24 h, Christ, Alpha 1-2 LD Plus, Lower Saxony, Germany). Table 1 shows the labeling of fifteen liposome nanoparticles prepared by varying the processing parameters: time and amplitude.

#### 4.2.2. Characterization of MXF-Free and MXF-Encapsulated Liposome Nanoparticles

In addition to the production of MXF-encapsulated liposomes, MXF-free liposomes were also synthesized for comparative analysis. Particle size (average size), PDI, and zeta potential values of resultant liposomes were figured out to unveil their stability capabilities. Dynamic light scattering (DLS) of MXF-free and MXF-encapsulated liposomes was measured to describe average particle size and PDI values. Zeta potential (ζ) measurements was conducted to design surface charge and describe possible interactions between drug and liposome [48]. Measurements of particle size, PDI, and zeta potential value were obtained by using Zetasizer (Nano series, Malvern Instruments Limited, Malvern, UK). FTIR analysis (PerkinElmer Spectrum One series FT-IR instrument, Version 5.0.1., Springfield, IL, USA) was conducted to characterize the chemical compounds. CA measurements (optical tensiometer and Attension Theta Contact Angle Meter, Phoenix, AZ, USA) assess the hydrophobicity of MXF-encapsulated constructs. SEM analysis for surface morphology description of MXF-free and MXF-encapsulated nanoparticles was performed using a PHILIPS XL 30 SFEG (York, UK), operated at an accelerating voltage of 5 kV. Moreover, fluorescent microscopy (Leica CTR600, Weltzar, Germany) observations were applied to measure particle size and confirm drug encapsulation in liposome nanoparticles. All measurements were made in triplicate.

#### 4.2.3. Preparation of MXF-Encapsulated Liposomes Enriched Alginate Nanocomposite Hydrogel Coatings on a Polymeric Substrate Material

MXF-free and MXF-encapsulated liposome-enriched alginate nanocomposite hydrogel coatings have been produced by using the following two different techniques: the TMJ device technique and the conventional micropipetting method, as illustrated in Figure 1. The TMJ device technique was constructed from a transparent polymer, polymethylmethacrylate (PMMA), using computer numerical control (CNC). The TMJ platform was made up of Teflon capillaries with a 200 µm internal diameter (ID) at the inlet (15 cm) and outlet (5 cm). The inert gas, nitrogen (N_2_), feeding capillary is attached vertically and controlled by a digital flow meter to obtain monodisperse microbubbles [49]. First, the polymer solution was attached to the system with horizontally positioned high-precision syringes (BD) with 10 mL volume connected to the syringe pump (IPS Series, Inovenso Pump Systemfs, Istanbul, Türkiye). To prepare the polymer solution, (0.5–5% *w*/*v*) of sodium alginate, 0.25% *w*/*v* of PEG, and 0.1–0.5% *w*/*v* of freeze-dried MXF liposome were combined in 10 mL of DI water and stirred until it was completely homogeneous.

Conventional MXF-encapsulated liposome-enriched alginate hydrogel was also performed to assess and compare in terms of the influence of the production techniques used. The conventional technique used to form alginate-based hydrogel coatings was micropipetting, which involves dropping the MXF-free and MXF-encapsulated liposome-enriched alginate nanocomposite hydrogel solution onto the polymeric substrate material by using a micropipette. Then, cross-linking was subsequently performed using calcium chloride (1% *w*/*v*). The resulting hydrogel coating constructs were lyophilized with a freeze-drier (at −55 °C, 1 mbar for 24 h), Christ, Alpha 1-2 LD Plus, Lower Saxony, Germany). 

Formation of alginate-based hydrogel coatings on polymeric substrate material by using the TMJ device technique was performed, as seen in Figure 1b. In this technique, by introducing the polymer solution into the system at a constant rate and gas pressure to exceed the surface tension of the solution, continuous bubble formation can be observed in the exit channel. Microbubbles that accumulate in the exit channel without agglomeration with each other are dripped onto the polymeric substrate material at a distance of approximately 1 cm. Calcium chloride (1% *w*/*v*) was then dropped onto the dome-like bubble cluster surface as a cross-linking agent for gelation by syringe. Samples were left at room temperature for 1 h at +4 °C and rinsed 3 times with distilled water to remove residual ions of calcium chloride. To facilitate the degassing process of the hydrogel, it will be subjected to an oven environment maintained at a pressure of 30 mbar for approximately one hour. The TMJ device procedure aims to promote the formation of uniformly porous structures interconnected by internal channels. After the gelling process, the samples will undergo freeze-drying in a lyophilizer for 24 h to enable comprehensive structural characterization.

### 4.3. Characterization of MXF-Encapsulated Liposome-Enriched Alginate Nanocomposite Hydrogels

#### 4.3.1. Fourier-Transform Infrared Spectroscopy (FTIR) Analysis of the Constructs

The chemical composition of the MXF-encapsulated liposome-enriched alginate nanocomposite hydrogel coating layers was determined by using a PerkinElmer Spectrum One series ATR-FTIR instrument (Version 5.0.1.). The scanning range was set between 4000 cm^−1^ and 400 cm^−1^.

#### 4.3.2. Morphological Characterization of the Resultant Alginate-Based Nanocomposite Hydrogel Coating Constructs

The cross-sectional view of the resultant MXF-encapsulated liposome-enriched alginate nanocomposite hydrogels was figured out by SEM investigation to examine the morphological features. MXF-encapsulated, liposome-free, and enriched alginate nanocomposite hydrogel coating constructs were captured with various magnifications. After being mounted on double-sided carbon tape, the hydrogel coating construct samples were curtained with a thin layer of gold to make them electrically conductive. On average, five images were captured for each sample at varying magnifications. The energy and intensity distributions of the X-ray signals produced by a concentrated electron beam on the sample were measured to conduct EDS microanalysis. The elemental mapping of the resulting samples was determined using the EDS.

#### 4.3.3. Analysis of Swelling Index

Swelling ratios of MXF-free and MXF-encapsulated liposome-enriched alginate nanocomposite hydrogels were determined by conventional gravimetric methods [50]. The dried (*W_d_*), preweighted nanocomposites were then immersed in 100 mL of DI water at room temperature for 120 min. At predetermined intervals, the samples were removed, gently wiped with filter paper to eliminate excess water, and subsequently reweighed to determine the swollen weight (*W_s_*) (*n* = 3). Then, the swelling index of nanocomposites was calculated using the following equation:

(1) $$Swelling\;Index\;\left(\%\right)=\frac{W_{s}-W_{d}}{W_{d}}\times 100$$

#### 4.3.4. Mechanical Measurements 

The mechanical properties of the MXF-free and MXF-encapsulated liposome-enriched alginate nanocomposite hydrogels were evaluated using an electro-mechanical universal tester (Instron 3382 Universal Testing Systems, Norwood, MA, USA) at room temperature. Each hydrogel sample, with dimensions of 12 mm in length, 4 mm in width, and 1.2 mm in thickness, was secured in the crossheads. The force-displacement data were collected using position control mode at a crosshead speed of 0.1 mm/s. Each hydrogel sample was tested three times to ensure accuracy, and the mean value and standard deviation were calculated. The force-displacement data were then converted to stress–strain to obtain the mechanical properties of the samples.

#### 4.3.5. In Vitro Drug Release Performance of the Resultant Constructs

In vitro drug release performance tests for the MXF-encapsulated liposomes and MXF-encapsulated liposome-enriched alginate nanocomposite hydrogel coating constructs were performed in water at a 37 °C temperature environment. First, the calibration curve of the drug MXF was plotted in order to assess the EE of liposomes. SL:MXF:2:1 liposomes (L1-L15) dissolved in DI water, and absorbance values were measured by using a UV-VIS spectrophotometer (SHIMADZU UV-3600, Kyoto, Japan) at $\lambda_{max}=290\;nm$ to unveil active pharmaceutical ingredients in drugs, where 10 measurements were taken and averaged for each liposome sample. UV spectroscopy was preferred for MXF detection due to its simpler instrumentation and ease of operation instead of complex analysis systems such as High-Performance Liquid Crystallography (HPLC). UV spectrophotometry was utilized as the analytical method due to the absence of spectral interference in the FTIR analysis and its benefits, including simplicity and quick result acquisition. By matching the results with the calibration curve, the EE of each sample was determined.

To evaluate MXF release from only bare liposome and MXF-encapsulated liposome nanoparticles, enriched alginate nanocomposites were produced by the following two different methods: (i) the conventional technique, micropipetting, and (ii) the TMJ device method. MXF-encapsulated liposome-enriched alginate nanocomposite hydrogels were placed in beakers containing 10 mL of DI water in a water bath at 37 ± 0.5 °C. At predetermined time intervals, samples of 2 mL were collected from the beakers and replaced with 2 mL of the fresh DI water to preserve the equilibrium conditions. For the quantitative analysis, collected samples were properly filtered and analyzed via UV spectrophotometer at λ_max_ of 290 nm. Experiments were performed in triplicate, and the mean values were utilized to determine the total cumulative drug concentration released. Using a standard calibration curve, the measured absorbance values were employed to calculate the cumulative concentration of MXF released at each time point.

#### 4.3.6. Evaluating Drug Release Profiles

To understand drug release mechanisms, the release profiles for all formulations were evaluated using different kinetic models, including the first-order, Higuchi, Korsmeyer–Peppas, and Hixson–Crowell models. Equations for all mathematical models are provided below.

The release of the drug can be represented for the first-order model as follows:

(2) $$\log C=\log C_{0}-K_{1}t/2.303$$

where $K_{1}$ is the first-order rate constant (time^−1^ or per hour), *C*_0_ is the initial concentration of the drug, and *C* is the percent of drug remaining at time *t*.

The Higuchi release model can be represented as follows:

(3) MtM∞=Kht12

where MtM∞ is the fraction of drug released at each time point (*t*), *M_t_* is the amount of drug released in time *t*, *M*_∞_ is the amount of drug released after time ∞, and *K_h_* represents the Higuchi release kinetic constant.

The Korsmeyer–Peppas model can be expressed as follows:

(4) MtM∞=Kkptn

(5) log⁡(MtM∞)=log⁡Kkp+nlog⁡t

Here, *M_t_*/*M*_∞_ is a fraction of drug released at time *t*, *M_t_* is the amount of drug released in time *t*, *M*_∞_ is the amount of drug released after time ∞, *n* is the diffusional exponent or drug release exponent, and *K_kp_* is the Korsmeyer release rate constant [51].

The equation about the Hixson–Crowell model is described in the following equation:

(6) $$W_{0}^{\frac{1}{3}}-W^{\frac{1}{3}}=k_{4}t$$

where *W*_0_ is the initial amount of active substance, *W* is the amount of undissolved active substance at *t*, *k*_4_ is the specific release constant, and *t* is the time [52].

#### 4.3.7. Statistics

Experiments were conducted in triplicate. The results are presented as mean ± standard deviation (SD) unless otherwise noted. *p* < 0.05 was considered to represent statistical significance. Statistical analysis was performed using Origin version 8.0.

## Figures and Tables

**Table 1:** SL:MXF:2:1 liposome formulation with changing parameters and labelled formulations.

Amplitude	Sonication Time (Min)				
(%)	2	4	6	8	10
**20**	L1	L2	L3	L4	L5
**40**	L6	L7	L8	L9	L10
**60**	L11	L12	L13	L14	L15

**Table 2:** Kinetics of in vitro drug release from MXF-encapsulated liposome nanoparticles layered coating form and MXF-encapsulated liposome-enriched alginate nanocomposite hydrogel coating constructs produced by the micropipetting method and the TMJ device technique for (**a**) the first 60 min and (**b**) between 120 and 1440 min.

(a)				
Formulation	R^2^ Value Observed by Applying Different Dissolution Models			
	First-Order	Higuchi	Korsmeyer–Peppas	Hixson–Crowell
MXF-encapsulated liposome (micropipetting method)	0.981	0.878	0.943 n = 0.294	0.977
MXF-encapsulated liposome (microfluidic method)	0.996	0.915	0.997 n = 0.864	0.995
Nanocomposite hydrogel (micropipetting method)	0.960	0.754	0.996 n = 1.282	0.966
Nanocomposite hydrogels (microfluidic method)	0.959	0.754	0.998 n = 1.370	0.962
(**b**)				
**Formulation**	**R^2^ Value Observed by Applying Different Dissolution Models**			
	**First Order**	**Higuchi**	**Korsmeyer–Peppas**	**Hixson–Crowell**
MXF-encapsulated liposome (micropipetting method)	0.676	−262.951	0.974	−135.750
MXF-encapsulated liposome (microfluidic method)	0.976	0.961	0.978 n = 0.441	0.967
Nanocomposite hydrogel (micropipetting method)	0.965	0.944	0.945 n = 0.515	0.994
Nanocomposite hydrogels (microfluidic method)	0.974	0.949	0.958 n = 0.556	0.924

## Data Availability

The original contributions presented in this study are included in the article/Supplementary Material. Further inquiries can be directed to the corresponding authors.

## Supplementary Materials

The following supporting information can be downloaded at: https://www.mdpi.com/article/10.3390/gels11060448/s1, Figure S1: FTIR spectra for pristine moxifloxacin, pristine lecithin, and SL:MXF:2:1 liposomes (soy lecithin + moxifloxacin) liposomes L1-L15.

## Abbreviations

The following abbreviations are used in this manuscript:

MXF	Moxifloxacin
TMJ	T-shaped Microfluidic Junction Device
EE	Encapsulation Efficiency
FTIR	Fourier-Transform Infrared Spectroscopy
SEM	Scanning Electron Microscopy
EDS	Energy-Dispersive Spectroscopy
PDI	Polydispersity Index
CA	Contact Angle
FDA	Food and Drug Administration
HPLC	High-Performance Liquid Chromatography
