# Supplementary material for: Engineering Moxifloxacin-Encapsulated Liposome-Enriched Alginate Hydrogel Films
Source: Gels. 2025 Jun 11;11(6):448. doi: 10.3390/gels11060448 (PMC12191549; doi:10.3390/gels11060448)
Supplement: Supplementary file 1 [file gels-11-00448-s001.zip › gels-3623189-supplementary.pdf]

Supplementary Data:

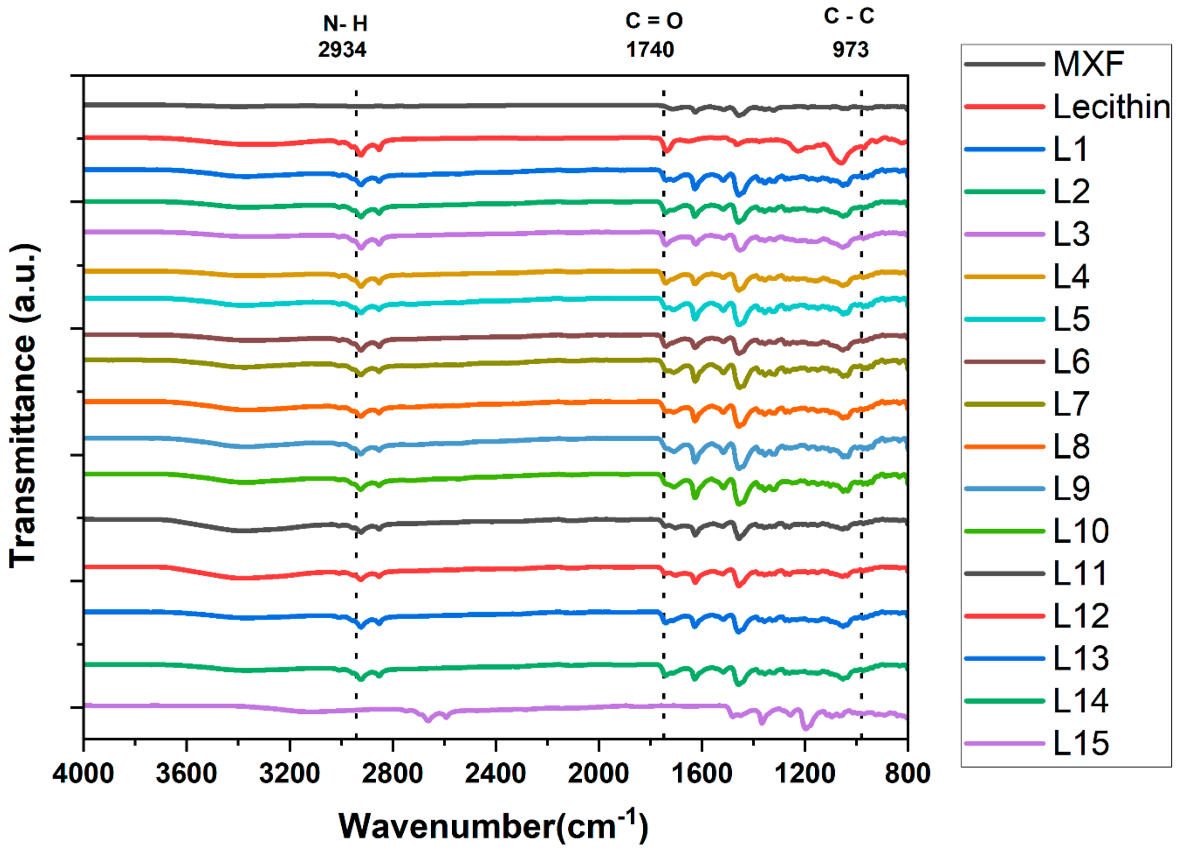

Figure S1 FTIR spectra for pristine moxifloxacin, pristine lecithin, and SL: MXF:2:1 liposomes (soy lecithin + moxifloxacin) liposomes L1-L15

Further confirmation of the drug-lipid interaction FTIR spectra of the liposomes achieved were measured and compared, as seen in Figure S1. The characteristic peaks in pure MXF spectra (Figure S1) were represented by the stretching vibrations C=O at 1735 cm^{-1}, N-H at 2949 cm^{-1}, and O-H at 3327 cm^{-1}. Soy lecithin showed peaks at 3671 cm^{-1} and 1703 cm^{-1} for the O-H and C=O stretching frequencies, respectively (Figure S1). Stretching vibrational peaks at 1170 cm^{-1} and 1062 cm^{-1} can be attributed to the C–O and C–C bonds. The primary absorption bands of lecithin and moxifloxacin hydrochloride were identified in the Fourier-transform infrared (FTIR) spectra of the liposomes produced, exhibiting no significant alterations (Figure S1, L1-L15). This observation suggests that there is no significant potential chemical interaction between the drug and the lipid components.
